# The cell senescence regulator p16 is a promising cancer prognostic and immune check-point inhibitor (ICI) therapy biomarker

**DOI:** 10.18632/aging.204601

**Published:** 2023-03-23

**Authors:** Zewei Tu, Xiaolin Wang, Huan Cai, Yilei Sheng, Lei Wu, Kai Huang, Xingen Zhu

**Affiliations:** 1 Department of Neurosurgery, The Second Affiliated Hospital of Nanchang University, Nanchang 330006, Jiangxi, P.R. China; 2 Jiangxi Key Laboratory of Neurological Tumors and Cerebrovascular Diseases, Nanchang 330006, Jiangxi, P.R. China; 3 Institute of Neuroscience, Nanchang University, Nanchang 330006, Jiangxi, P.R. China; 4 JXHC Key Laboratory of Neurological Medicine, Nanchang 330006, Jiangxi, P.R. China; 5 The Second Clinical Medical College of Nanchang University, Nanchang 330006, Jiangxi, P.R. China; 6 Department of Medical Ultrasonics, Integrated Chinese and Western Medicine Hospital of Jiangxi Province, Nanchang 330006, Jiangxi, P.R. China; 7 The HuanKui Medical College of Nanchang University, Nanchang 330006, Jiangxi, P.R. China

**Keywords:** CDKN2A, cell senescence, cancer prognosis, biomarker, immunotherapy

## Abstract

Cyclin-dependent kinase inhibitor 2A (CDKN2A) encodes the cell senescence regulator protein p16. The expression of p16 raises in cell senescence and has a nuclear regulation in cell aging. Meanwhile, it's also reported to inhibit the aggression of several cancers. But its clinical application and role in cancer immunotherapy needs further investigation. We collected the transcriptional data of pan-cancer and normal human tissues from The Cancer Genome Atlas and the Genotype-Tissue Expression databases. CBioPortal webtool was employed to mine the genomic alteration status of CDKN2A across cancers. Kaplan-Meier method and univariate Cox regression were performed for prognostic assessments across cancers, respectively. Gene Set Enrichment Analysis is the main method used to search the associated cancer hallmarks associated with CDKN2A. TIMER2.0 was used to analyze the immune cell infiltration relevance with CDKN2A in pan-cancer. The associations between CDKN2A and immunotherapy biomarkers or regulators were performed by spearman correlation analysis. We found CDKN2A is overexpressed in most cancers and exhibits prognosis predictive ability in various cancers. In addition, it is significantly correlated with immune-activated hallmarks, cancer immune cell infiltrations and immunoregulators. The most interesting finding is that CDKN2A can significantly predict anti-PDL1 therapy response. Finally, specific inhibitors which correlated with CDKN2A expression in different cancer types were also screened by using Connectivity Map (CMap) tool. The results revealed that CDKN2A acts as a robust cancer prognostic and immunotherapy biomarker. Its function in the regulation of cancer cell senescence might shape the tumor microenvironment and contribute to its predictive ability of immunotherapy.

## INTRODUCTION

Immunotherapy of tumors by targeting immune checkpoint receptor has been the new trend and has a significant effect on cancer curing. However, current immune checkpoint blockade doesn't work for every cancer patient, and a great number of patients don't achieve the expected curative effect or even respond at all to the existing immunotherapy agents. Thus, searching for a new, effective immunotherapy biomarker or target is desired and necessary, to cut down tumor mortality and reduce ineffective treatment.

CDKN2A gene (chromosome 9p21) encoding the protein p14ARF and p16INK4a, also known as p16, formed through alternative exon usage [1]. Deletion, point mutation, and/or promoter methylation can cause damage to CDKN2A gene function, which, in turn, can lead to uncontrolled cell proliferation, giving rise to the evolution and progression of the tumor [2]. The frequency of CDKN2A loss-of-function has a wide range from 20% to 85% in various cancers [1]. The genomic alterations of p16 contain point-mutation, promoter hypermethylation, homozygous deletion as well as loss of heterozygosity (LOH) [3]. The homozygous deletion and promoter hypermethylation are the most frequent genetic alteration types which caused p16 inactivation [1]. Moreover, Mutation and deletion of CDKN2A are major genetic changes discovered in melanoma cell lines [4].

The protein of p16 can inhibit cyclin-dependent kinase 4 (CDK4) and CDK6 and p16 plays vital role as a tumor suppressor in various human malignant cancers, including colon cancer. When activated, p16 protein consists of four anchor protein repeat sequences [5]. Once CDK6 bonds to the cavity of p16, its catalytic cleft is exposed to p16, inducing the interaction of D84 of p16 to R31 of CDK6 that may result in decreased kinase activity. Moreover, p16 protein can inhibit the function of CDK4/6 via impairing interaction with cyclin D [3].

The CDKN2A gene generates four transcriptional variants by utilizing alternative exons: p16INK4A, p12, p14ARF and p16γ [3]. p14ARF activates the p53 pathway, while p16 cuts off the progression of the G1/S transition by inhibiting the phosphorylation of Rb. The interruption of these pathway controls the progression of a variety of cancers [6]. p16INK4A acts as a tumor- suppressor by binding to CDK4/6 and thus restrict the cell cycle to enter the G1/S phase by cutting off interaction of CDK4/6 and cyclin D1, and later phosphorylation process of retinoblastoma protein (RB1) [7]. CDK2 (p12) protein plays a key role in the late G1-to-S phase transitional process through phosphorylating Rb protein while the phosphorylated Rb protein could activate gene transcription regulated by E2F, which is necessary for DNA replication. In addition, p12^CDK2-AP1^ was also shown to be related to the polymerase *a* -primase and block the initial stages of DNA replication [8]. p16γ is highly expressed at transcription and translation levels in primary T-ALL patients and neuroblastoma cell lines, and at low levels in other samples of primary T-ALL and B- lineage -ALL patients expressing p16INK4A. p16γ act by interacting with CDK4 to inhibit CDK4 kinase activity and cell proliferation [9].

CDKN2A is the second most commonly deactivated tumor suppressor gene [10] and it plays a prominent role in many common malignant tumors. Many studies have interpreted and studied the important role of p16 in the occurrence and development of tumors from the perspective of different types of tumors. For instance, deleted CDKN2A gene locus and loss of expression exist in many chordoma cell lines [11]. Moreover, it is indicated that CDKN2A might also drive the pancreatic cancer initiation and progression, because somatic mutations of CDKN2A are widespread in about 95% of pancreatic cancer patients [12]. Besides, Abnormal regulation of CDKN2A/p16 gene often occurs in that development of oral squamous cell carcinoma (OSCC) [13].

Tumor immunotherapy is the main trend of tumor treatment nowadays, however, there are few studies on whether the important anti-cancer factor p16 participates in and plays a role in tumor immunotherapy. For instance, in highly differentiated and dedifferentiated liposome, with or without the combination of CDK4 and MDM2, p16 nuclear immunopositivity is of diagnostic significance and value [14]. Besides, in the context of CDKN2A deletion, one of the abnormal signal nodes of pancreatic cancer is characterized by increased ability of HGFR and EGFR, and neuropilin 1, CD44 and β1 integrin get an increased expression [15].

At present, CDKN2A has been deeply studied in genomics and signaling pathway of specific malignant tumors, but its research on specific types of tumor immunity is less, and the research on pan-cancer immunity is even shallower. CDKN2A is one of the most reported tumor suppressors in cancer progression and associated with immune evasion by T cell killing. But the role of CDKN2A in cancer immune infiltrations and immunotherapy response prediction is not clear. Therefore, this study attempted to investigate whether CDKN2A can be used as a robust tumor marker and play a role in pan-cancer immunotherapy, thereby providing some clues for tumor immunotherapy strategy.

## MATERIALS AND METHODS

### Data source

To analyze the CDKN2A mRNA expression in human tissues, we collected the mRNA expression and clinical materials that derives from Genotype-Tissue Expression (GTEx) datasets and the TCGA pan-cancer cohort from the UCSC Xena database (https://xenabrowser.net/datapages/). We used the webtool of cBioPortal for Cancer Genomics (http://cbioportal.org) to detect the genomic alteration frequency of CDKN2A in the 33 cancer types. we assessed the expression of CDKN2A protein at the subcellular level through The HPA (Human Protein Atlas) (HPA: https://www.proteinatlas.org/) database. We investigated the CDKN2A protein interaction information by the ComPPI database (compartmentalized protein-protein interaction--http://comppi.linkgroup.hu) [16]. Supplementary Table 1 illustrates the abbreviations of cancers we investigated.

### Prognosis analysis of CDKN2A across cancers

We used the UCSC Xena database (https://xenabrowser.net/datapages/) to investigate prognosis data of CDKN2A including overall-survival (OS), progression free interval (PFI), disease specific survival (DSS) and disease-free interval (DFI). We assessed the prognostic role of CDKN2A for the specific prognosis type in cancer through univariate Cox regression and Kaplan Meier model. The univariate Cox regression adopted the continuous variable of CDKN2A expression data materials. We performed Kaplan-Meier curves analysis on bivariate CDKN2A expression levels and its cutoff was selected by the “surv-cutpoint” function of “survminer” R package (0.4.9). We calculated hazard ratio (HR) with 95% confidence interval (95%CI) and the log-rank p value of K-M method. We described the results as a heat map.

### Identification of differential expression genes between low- and high-CDKN2A subgroup

In order to determine the differentially expressed genes between the low CDKN2A subgroup and the high CDKN2A subgroup in each cancer, the cancer patients were ranked according to the CDKN2A mRNA expression. The first 30% patients were defined as the high CDKN2A subgroup and the last 30% patients were defined as the low CDKN2A subgroup. By using “limma” R package [17] for differential expression analysis, we acquired log2 (multiple change) and adjusted P value of each gene in each cancer type. Genes with p- value < 0.05 were defined as differentially expressed genes (DEGs). The DEGs between the low and high CDKN2A subgroups of each cancer was summarized in Supplementary Table 2.

### Gene set enrichment analysis

We downloaded the “gmt” file of hallmark gene set from Molecular Signatures Database (MSigDB, https://www.gsea-msigdb.org/gsea/index.jsp) [18] and computed false discovery rate (FDR) and the normalized enrichment score (NES) between low- and high-CDKN2A cancer group about their biological process in every cancer type. The GSEA was investigated by the R package “clusterProfiler” [19] and its results were detected in the bubble chart described by R package “ggplot2”.

### Immunotherapy prediction analysis

We used spearman correlation analysis to demonstrate the statistical correlation between CDKN2A and some immunotherapeutic biomarkers such as microsatellite instability (MSI), tumor mutation burden (TMB), and other immune checkpoint genes in pan-cancer. We obtained two treatment cohorts with immune checkpoint blockade (ICB) to analyze the predictive power of CDKN2A for immunotherapy response, the IMvigor210-urological cohort [20] and GSE9061-melanoma cohort.

### Single cell analysis and compounds correlating with CDKN2A in pan-cancer

The expression of CDKN2A in single cell level of cancer samples were investigated in the website of Tumor Immune Single-cell Hub (TISCH, http://tisch.comp-genomics.org/) [21]. The Connectivity Map (CMap) could connect diseases with effective drugs as the new implement for Biomedical Science research. We used CMap (https://portals.broadinstitute.org/cmap/) [22] to confirm the relationships between CDKN2A expression levels and specific inhibitors in pan-cancer. We visualize the heatmap and utilize the webtool. Associated enriched compounds were summarized in Supplementary Table 3.

### Sample collection and Western bolt

Seven LGG samples with relative adjacent tissues were resected from inpatients who were under treatment in the Neurosurgery Department of The Second Affiliated Hospital of Nanchang University (NCUSAH) in 2021. The tumor excisions were stored in liquid nitrogen after excising from LGG patients. Informed consents were acquired from inpatients enrolled in this study. The usage of clinical excisions was consented by the Medical Ethics Committee of NCUSAH. The processes of clinical samples collection and usage were in strict accordance with the guideline. The rabbit polyclonal P16-INK4A antibody (10883-1-AP, Proteintech, China) and rabbit polyclonal anti-beta-Tubulin (10068-1-AP, Proteintech) were purchased and used for western blot assay in a diluent concentration of 1:1000 and 1:2000, respectively. The immunoblot assay was completely consistent with the workflow described in the previous study we conducted [23].

### Cell culture and siRNA transfection

SW1088 cell line was purchased from the American Type Culture Collection (ATCC) and cultured with Leibovitz’s L-15 Medium (Gibco), added with 10% fetal bovine serum (FBS, Gibco) in the normal fresh air at 37° C and 100% humidity.

Pre-designed three types of CDKN2A siRNA and negative control siRNA (Sheweisi Company, Tianjin, China) were transfected into SW1088 cells using lipo3000 reagent (ThermoFisher, USA) as instructions. The subsequent assays were conducted after 24h cell transfections with siRNAs.

### CCK8 and cell colony formation assays

The cell proliferation ability was evaluated by CCK8 cell proliferation assay and colony formation assay. Two thousand SW1088 cells, transfected with siRNAs, were calculated and spread into the one well of the 96 well plate for 24h cultured. And CCK8 agent (Beyotime, China) was added as instruction, and cultured for 1, 2, 3 and 4 days. The absorbency of each well was tested by microplate reader at the time point described above.

Five hundred SW1088 cells with transfection were prepared and spread into one well of the 6-well plate. After culturing for two weeks, the cell cultured medium was removed and washed with phosphate buffer solution (PBS), then fixed with 4% paraformaldehyde for 1 hour and 0.1% crystal violet solution for 6 hours. The cell colony number of each well were calculated using ImageJ software.

### Immunohistochemistry (IHC) staining

Immunohistochemistry (IHC) staining of p16 protein in the six paired LGG and adjacent tissues were conducted as a previous study [24]. The 1:1000 concentration dilution of rabbit P16-INK4A Polyclonal antibody (10883-1-AP, Proteintech, China) was used as the primary antibody to stain the p16 protein in these tissues.

### Statistical analysis

In order to compare the expression level of CDKN2A between normal and tumor tissues, we used Wilcoxon rank sum test for statistical significance calculation. Paired t-test was utilized to assess the statistical significance of CDKN2A protein expression in clinical LGG tissues and adjacent tissues. Kaplan-Meier method and univariate Cox regression analysis were used to evaluate the prognosis of CDKN2A expression in every cancer. Spearman correlation analysis was used to evaluate the statistical relationships between CDKN2A and else factors. Chi-square test was used to calculate the statistical significance in order to compare the proportion of ICI-therapy responder and non-responders in cancer subgroups with low CDKN2A and high CDKN2A. Student’s t-test was used to compare the cell colony number and optical density (OD) values of CCK8 assay between two groups. All the codes used in this study were uploaded in the Github (https://github.com/tzw2019/Pancancer-CDKN2A).

### Data availability statement

The original data used in this project can be downloaded in the UCSC (https://xenabrowser.net/datapages/) website.

## RESULTS

### Basic information of CDKN2A

**Figure 1:**
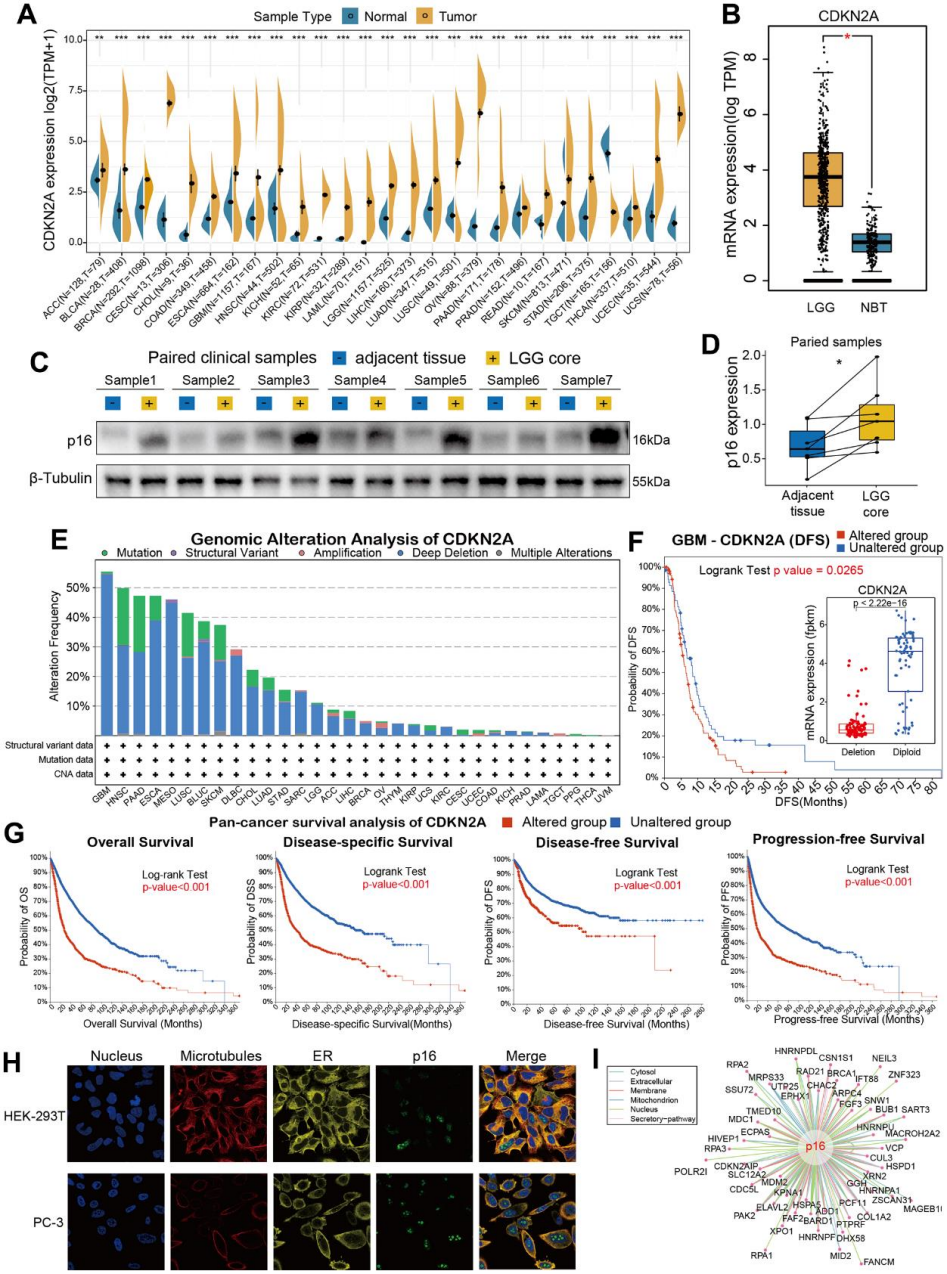
**Basic information for CDKN2A.** (**A**) The expression level of CDKN2A between tumor and normal tissue of each cancer based on the integration of data from the TCGA and GTEx datasets. (**B**)The CDKN2A expression in LGG and normal brain tissue. (**C**) The western blot assay showed the gray gels of proteins of LGG and adjacent tissues. (**D**) Quantitative analysis showed the upregulation of CDKN2A in LGG samples compared with adjacent tissues. (**E**) Analysis of the frequency of CDKN2A changes in pan-cancer studies based on the cBioPortal database. (**F**) CDKN2A expression levels and DFS prognosis in GBM patients with CDKN2A altered and unaltered. (**G**) Pan-cancer survival of CDKN2A. (**H**) Immunofluorescence images and fusion images of CDKN2A protein in nuclei, endoplasmic reticulum, and microtubules in A-431 and U251 cell lines. (**I**) The protein-protein interaction (PPI) network presents a protein interacting with CDKN2A. Asterisks indicate statistical p-values (ns p>0.05, *p<0.05, **p<0.01, and ***p<0.001).

In order to analyze the fundamental information of CDKN2A in cancers, we used the transcriptomic data obtained from TCGA and GTEx databases to assess the mRNA expression levels of CDKN2A in cancers compared with human normal tissues. Almost all of TCGA cancers have a higher expression level of CDKN2A than normal tissues, including ACC, BLCA, GBM, HNSC, KICH, KIRC, KIRP, LAML, LGG, LIHC, LUAD, LUSC, OV, PAA, BRCA, CESC, CHOL, COAD, ESCA, PRAD, READ, SKCM, STAD, THCA, UCEC, UCS. It. While, only TGCT has a lower CDKN2A expression than normal tissues (Figure 1A). Besides, the expression of CDKN2A in normal human samples (Supplementary Figure 1A) and cancer cell lines (Supplementary Figure 1B) were also presented in our study. Particularly, we found the mRNA expression of CDKN2A in LGG is significantly increased (Figure 1B) compared with normal brain tissues, which caught our interests. To verify whether aberrant protein expression of p16 exists in LGG, we performed western blot assay to detect the protein of p16 in seven pairs of LGG samples with related adjacent tissues (Figure 1C, original data were shown in Supplementary Figure 2), and quantitative results showed that the expression of p16 is upregulated in LGG cores compared with adjacent tissues (Figure 1D). Then we conducted genomic alteration analysis of CDKN2A and found the alterations frequency of CDKN2A across pan-cancer are generally high (Figure 1E), alteration frequencies of 14 cancers are more than 10%. More interestingly, deep deletion frequency of CDKN2A is most high in most instances. The high deletion frequency of CDKN2A has been shown to be associated with a variety of tumors such as melanoma, lung cancer, head and neck cancer, pancreatic cancer, breast cancer, osteosarcoma, ovarian cancer [25]. In particular, the most frequently altered cancer type was GBM, exceeding 50% GBM patients and deep deletion is the highest frequent alteration (Figure 1F). Then we conducted pan-cancer survival analysis of CDKN2A and we found that the OS, DSS, DFS, PFS time of the CDKN2A altered group are all shorter than unaltered group (Figure 1G, all the four p-value <0.001, log-rank test). Moreover, immunofluorescence (IF) images showed that CDKN2A protein was mainly distributed in the nucleus of HEK-239T and PC-3 cell lines (Figure 1H, original data were shown in Supplementary Figure 3). Lastly, we established the protein-protein interaction (PPI) network by exploiting the interaction data acquired from the ComPPI website. The network shows that the subcellular localizations information of protein p16 were mainly distributed in extracellular, secretory pathway, cytosol, mitochondria, nucleus and membrane subcellularly (Figure 1I), and interactor proteins were shown as depicts. To illustrate the expression distribution of CDKN2A in tumor microenvironment, we also visualized the expression of CDKN2A in different cells using the TISCH webtool (Supplementary Figure 4). The results showed that CDKN2A was mainly expressed in the malignant cells in tumor microenvironment, which indicated the important role of p16 in cancer cells.

### Prognostic analysis of CDKN2A in pan-cancer

**Figure 2:**
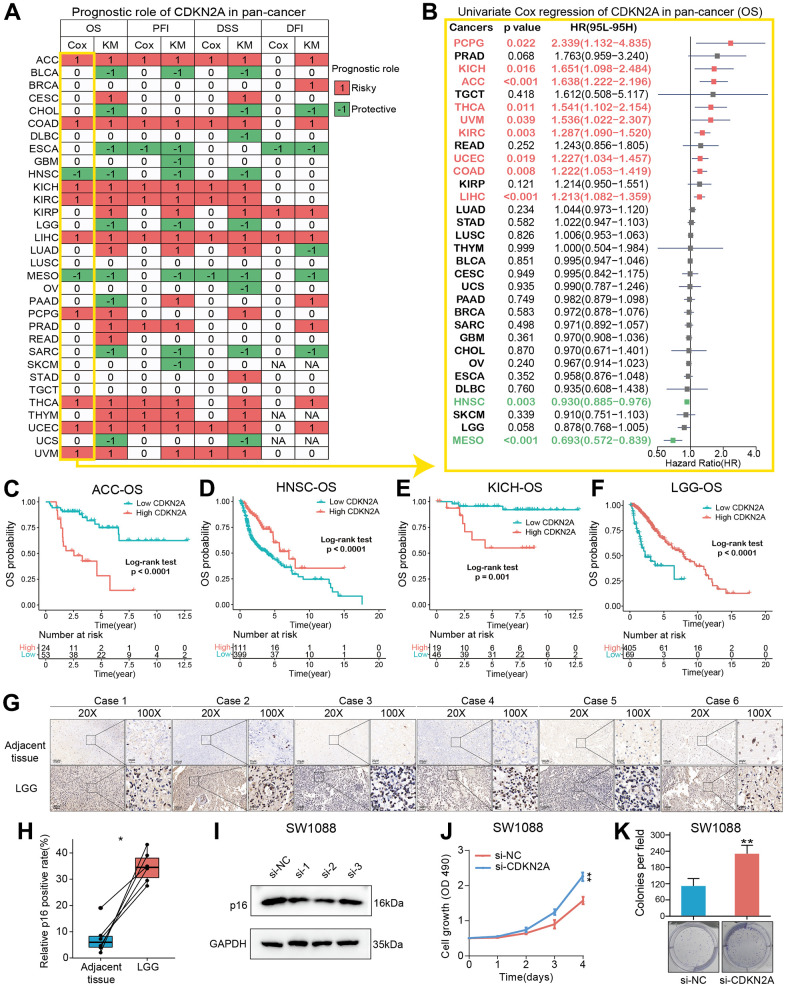
**Prognostic roles of CDKN2A in pan-cancer.** (**A**) Summary of the correlations between CDKN2A expression and overall survival (OS), disease-free interval (DFI), disease-specific survival (DSS) and progression-free interval (PFI) of cancer patients according to univariate Cox regression and Kaplan-Meier model. Red indicates that CDKN2A is the risk factor affecting prognosis of cancer, and green indicates a protective factor. Only p value < 0.05 are colored. (**B**) Through univariate Cox regression method, forest map shows the prognostic role of CDKN2A in cancer. The red cancer type indicates that CDKN2A is a statistically significant risk factor. (**C**–**F**) Kaplan-Meier total survival curve of CDKN2A in ACC-OS (**C**), HNSC-OS (**D**), KICH-OS (**E**) and LGG-OS (**F**). (**G**) Immunohistochemical images showed the expression and location of p16 protein in six pairs of LGG and adjacent tissues. (**H**) Paired-t test showed the statistical significance of p16 positive rate of the IHC results. (**I**) Western blot validated the knock-down effects of the siRNAs in SW1088 cells. (**J**, **K**) CCK8 cell proliferation analysis (**J**) and cell colony formation assay (**K**) showed the discrepancy of cell proliferation abilities between SW1088 cells with or without CDKN2A knock-down.

We constructed a heatmap to show the prognosis analysis results (including univariate Cox and Kaplan-Meier methods) of CDKN2A in pan-cancer, and we found that CDKN2A has strong prognostic correlations with most cancers except TGCT (Figure 2A). Particularly, the OS analysis results suggested that CDKN2A plays risky roles in the prognosis of tumors such as AAC, COAD, KICH, KIRC, LIHC, PCPG, THCA, UCEC and UVM. While CDKN2A plays a protective role in the prognosis of HNSC patients. Since the OS survival outcome endpoint included non-cancer deaths, we performed the disease-specific survival (DSS) analysis that was more relevant to the efficacy of cancer treatment. The results of DSS analysis were basically consistent with OS analysis, which both indicated that CDKN2A played a risky role in the prognosis of the above cancers. Results of disease-Free Interval (DFI) and Progression-Free Interval (PFI) analysis were also examined to adequately demonstrate that CDKN2A is a risk factor for most cancer types and is significantly associated with the prognosis of cancer. In addition, results from OS, DSS, PFI, and DSS all indicated that CDKN2A was a protective factor for ESCA, MESO and SARC.

To further understand how CDKN2A could be a predictive biomarker for cancer patient prognosis, we performed the univariate Cox regression to investigate the prognosis of 32 TCGA cancer types. Results from forest plot showed that the down-regulation of CDKN2A expression was significantly associated with OS prolongation in the ACC (HR = 1.638[95%CI, 1.222-2.196], p<0.001), KICH (HR = 1.651[95%CI, 1.098-2.484], p = 0.016), HNSC (HR = 0.930[95%CI, 0.885-0.976], p = 0.003), MESO (HR = 0.693[95%CI, 0.572-0.839], p <0.001) (Figure 2B). Kaplan-Meier curve analysis on ACC and KICH were showed that the higher CDKN2A expression was associated with poor survival rate (Figure 2C, 2E), indicating that CDKN2A is a biomarker for the prognosis of ACC and KICH. Further Kaplan-Meier survival analysis also confirmed that higher CDKN2A expression in HNSC and LGG (Figure 2D, 2F) was associated with better prognosis. The loss of CDKN2A is known to be associated with OS shortening of HNSC and a shorter repetition-free survival time in HNSC [26]. To investigate the biological functions of CDKN2A in LGG, in which cancer type the CDKN2A could predict the prognosis, we firstly detected the expression of p16 in clinical LGG samples and designed siRNAs to target the CDKN2A expression. As the immunohistochemical images showed (Figure 2G, original data were shown in Supplementary Figure 5), the p16 expression was significant upregulated in LGG samples compared with adjacent tissues (Figure 2H), which was consistent with the transcriptomic analysis in the Figure 1A. We then validated the knock-down efficiency of the three designed siRNAs in SW1088 cells, the number 2 siRNA showed most effective knock-down effect (Figure 2I, original data were shown in Supplementary Figure 6). Then CCK8 cell proliferation analysis and cell colony formation assay were conducted to investigate the biological functions of CDKN2A in LGG cells. Both of the two assays showed that knock-down of CDKN2A could results an enhanced proliferative ability in SW1088 cells (Figure 2J, 2K), which showed the CDKN2A also act as a tumor suppressor in LGG cells.

### GSEA of CDKN2A in pan-cancer

**Figure 3:**
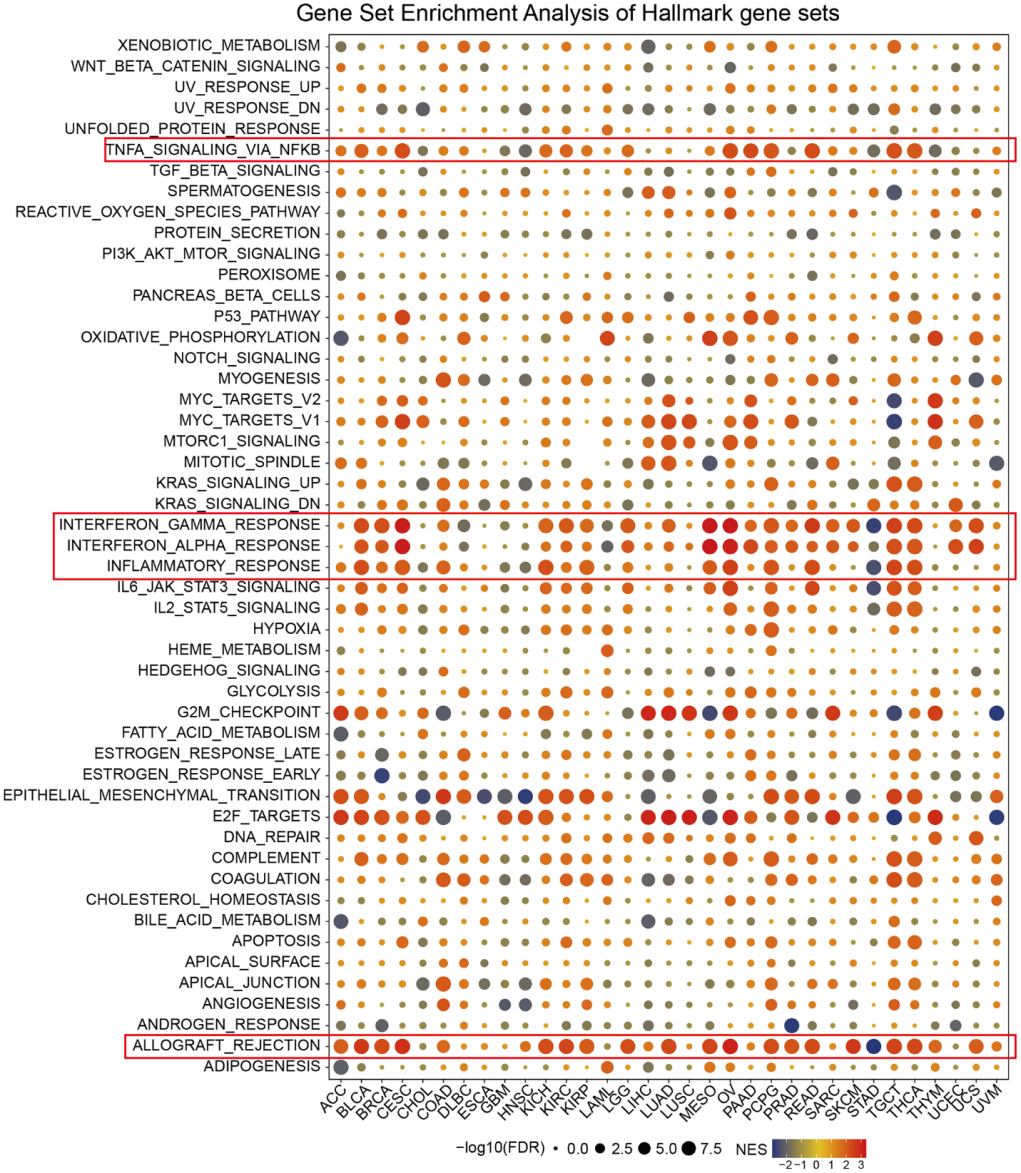
**Enrichment analysis of CDKN2A marker gene set in pan-cancer.** The circle size represents the FDR value of the enrichment term about each cancer. The color signifies the normalized enrichment score (NES).

We carried out gene set enrichment analysis (GSEA) of hallmark gene sets in different tumor types to find out the CDKN2A associated cancer characteristics. Interestingly, we found that CDKN2A expression was significantly associated with tumor immune-related pathways such as the TNFA signaling pathway via NFKB, IFN-α response, IFN-γ response, allograft rejection pathway and inflammatory response, especially in BLCA, CESC, MESO, OV, TGCT and THCA (Figure 3). The tumor microenvironment is mainly composed of the vasculature, extracellular matrix (ECM) [27], non-malignant cells around, and a complex network of signaling molecules, such as growth factors, chemokines, and cytokines [28]. Analysis of these data above revealed a potential association between CDKN2A expression and immune activation in the tumor microenvironment (TME). On the other hand, epithelial-mesenchymal transition markers have significantly high expression in the high CDKN2A subgroup, such as BLCA, BRCA, CESC, MESO, OV, TGCT and THCA. Previous studies have clarified that EMT is closely related to the occurrence, metastasis as well as drug resistance of tumors [29], suggesting that CDKN2A might play an indispensable role in the occurrence and development of tumors through associating with EMT. Moreover, E2F-targets are also closely related to the expression of CDKN2A in cancer. In general, these results indicated that the high expression of CDKN2A is related to the immunologic activation of cancer and may provide some clues for further research on the function and role of CDKN2A in the occurrence and development of cancer.

### TIMER immune cell Infiltration analysis

**Figure 4:**
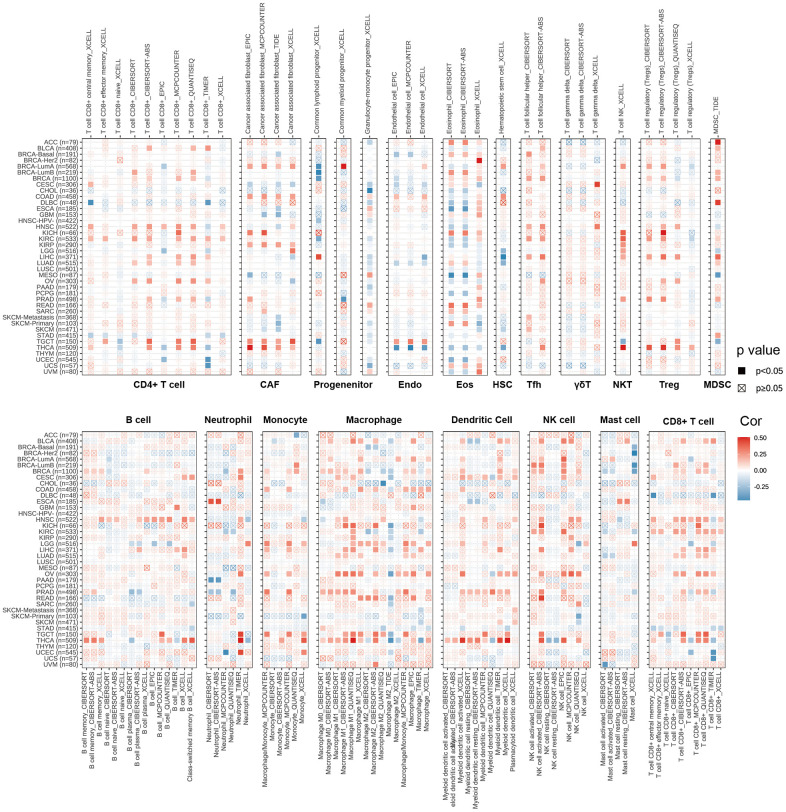
**The relations between CDKN2A expression and infiltration levels of different kinds of immune cell in cancer.** Red is a positive correlation and blue is a negative correlation.

CD4+T cells, CAF, MDSC, neutrophils and macrophages have been shown to play key roles in tumor immunotherapy [30, 31]. The immune cells are a part of the tumor microenvironment, such as T cells, Tregs, B cells, MDSCs, NK cells, DCs [32]. We used the pan-cancerous immunocyte infiltration data from the TIMER2 database for Spearman correlation analysis to mine the correlations between CDKN2A expression and immunocyte infiltrations to further analyze the relationship between CDKN2A expression and tumor immunity. The results showed the correlations between CDKN2A expression and different infiltration levels of CD4+ T cells, CAF, progenitors, NK T cells, Tregs, B cells, Endo, Eos, HSC, Tfh, γ/δT, neutrophils, monocytes, macrophages, dendritic cells, NK cells, Mast cells and CD8+ T cells in pan-cancer (Figure 4). We uncovered that CDKN2A was positively correlated with infiltration degree of CD4+ T cells, NKT cells, Tregs, MDSC, neutrophils and macrophages in most TCGA cancers. Besides, we also uncovered that CDKN2A was negatively correlated with infiltration degree of CAF, progenitors, Endo and Eos. From another point of view, CDKN2A is positively correlated with the level of infiltration of most immunocytes such as B cell, NK cell, Mast cell, CD8+ cell, Dendritic cell, neutrophils, monocytes and macrophages in the TGCT and THCA. Our research results indicate that CDKN2A may affect the occurrence, prognosis and treatment of tumors by associating to immune cells.

### Relationships between CDKN2A and immune regulators, TMB, and MSI

**Figure 5:**
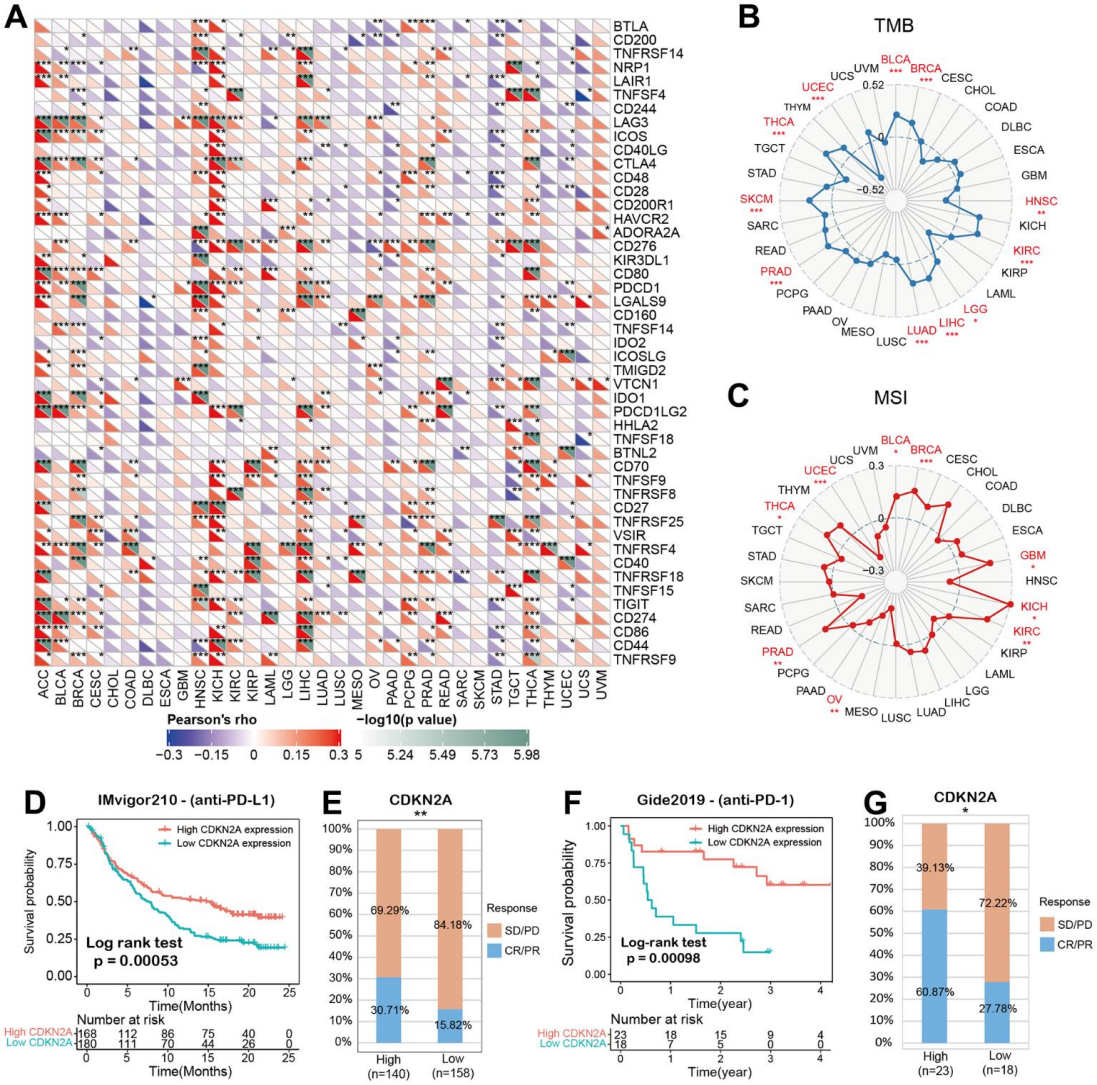
**CDKN2A predicts the immunotherapy response of cancer patients.** (**A**) spearman correlation thermogram shows the correlation between CDKN2A expression in pan-cancer and immune regulatory factors. Red and blue represent positive correlation and negative correlation, respectively. (**B**) The correlation between CDKN2A expression and tumor mutation in cancer. (**C**) Correlation between CDKN2A expression and microsatellite instability in pan cancer. (**D**, **E**) Kaplan-Meier curve (anti-PD-L1, urology) of patients with low CDKN2A and high CDKN2A in IMvigor210 cohort; proportion of urological tumor patients in subgroups of low CDKN2A and high CDKN2A in imvigor210 cohort which responded to PD-1 treatment. (**F**, **G**) Kaplan-Meier curves (anti-PD-L1, melanoma) of patients with low and high CDKN2A in GSE91061-melanoma; proportion of melanoma patients who responded to PD-1 treatment in subgroups of low and high CDKN2A in GSE91061-melanoma. The asterisk symbolizes the statistical p value (*p<0.05, ** p<0.01, *** p<0.001).

We analyzed the associations between CDKN2A and 47 immune factors in pan-cancer (Figure 5A). We uncovered a strong positive correlation between CDKN2A and most immunomodulatory factors in ACC, KICH, and LICH. Besides, CDKN2A was strongly and negatively correlated with most immunomodulatory factors in CHOL, DLBC, ESCA. The tumor mutation burden (TMB) represents other biomarkers which may predict the response to tumor immunotherapy [33]. TMB has been proved to predict survival for different types of cancer [34]. Besides, microsatellite instability (MSI) has also been shown to be a predictive biomarker for tumor immunotherapy [35]. To investigate the role of CDKN2A in predicting the potential response of immune checkpoint inhibitors (ICIs) therapy, we further evaluated the association of CDKN2A expression with TMB and MSI. Positive correlations with TMB were found in LGG, COAD, BLCA, THYM, THCA, STAD, SKCM, and PAAD (Figure 5B). In addition, there were positive correlations between CDKN2A expression and MSI in BLCA, BRCA, GBM, KICH, KIRC, PRAD and THCA and negative correlations found in UCEC and OV (Figure 5C). Our results suggest that CDKN2A has the potential to infer the efficacy of ICIs in corresponding cancers.

### The predictive role of CDKN2A in cancer immunotherapy

Immune checkpoint inhibitors (ICIs), for example, inhibitors targeting programmed cell death 1 (PDCD1) protein and its ligand protein of PD-L1, have made a significant contribution to tumor immunotherapy [36]. For example, anti-PD-L1 antibody has been the focus of many studies recently [37–39]. Based on the above results, we then analyzed the predictive role of CDKN2A, as an immunotherapy response biomarker, in cancer cohort treated with ICIs. The relationships between CDKN2A and the anti-PDL1 therapeutic response in patients with urological neoplasms suggests that patients with higher CDKN2A expression levels showed better prognosis (Figure 5D).

Checkpoint programmed cell death–1 and its ligand (PD-1 and PD-L1) inhibit the effector T lymphocytes and eventually result in tumor immune escape [40]. Blocking these immune checkpoints has become a hot topic and direction of tumor immunotherapy in recent years. In the IMvigor210 cohort of urological tumors, the response rate against PD-L1 in patients with high CDKN2A expression was 30.71%, significantly higher than 15.82% in patients with low CDKN2A expression (Figure 5E). Similar findings were also observed in melanoma patients treated with anti-PD-1 therapy (Figure 5F, 5G). These data demonstrate the potential ability of CDKN2A in the prediction of immunotherapy response and suggest that CDKN2A is a hopeful biomarker in cancer immunotherapy.

### Connectivity map (CMap) analysis of CDKN2A in pan-cancer

**Figure 6:**
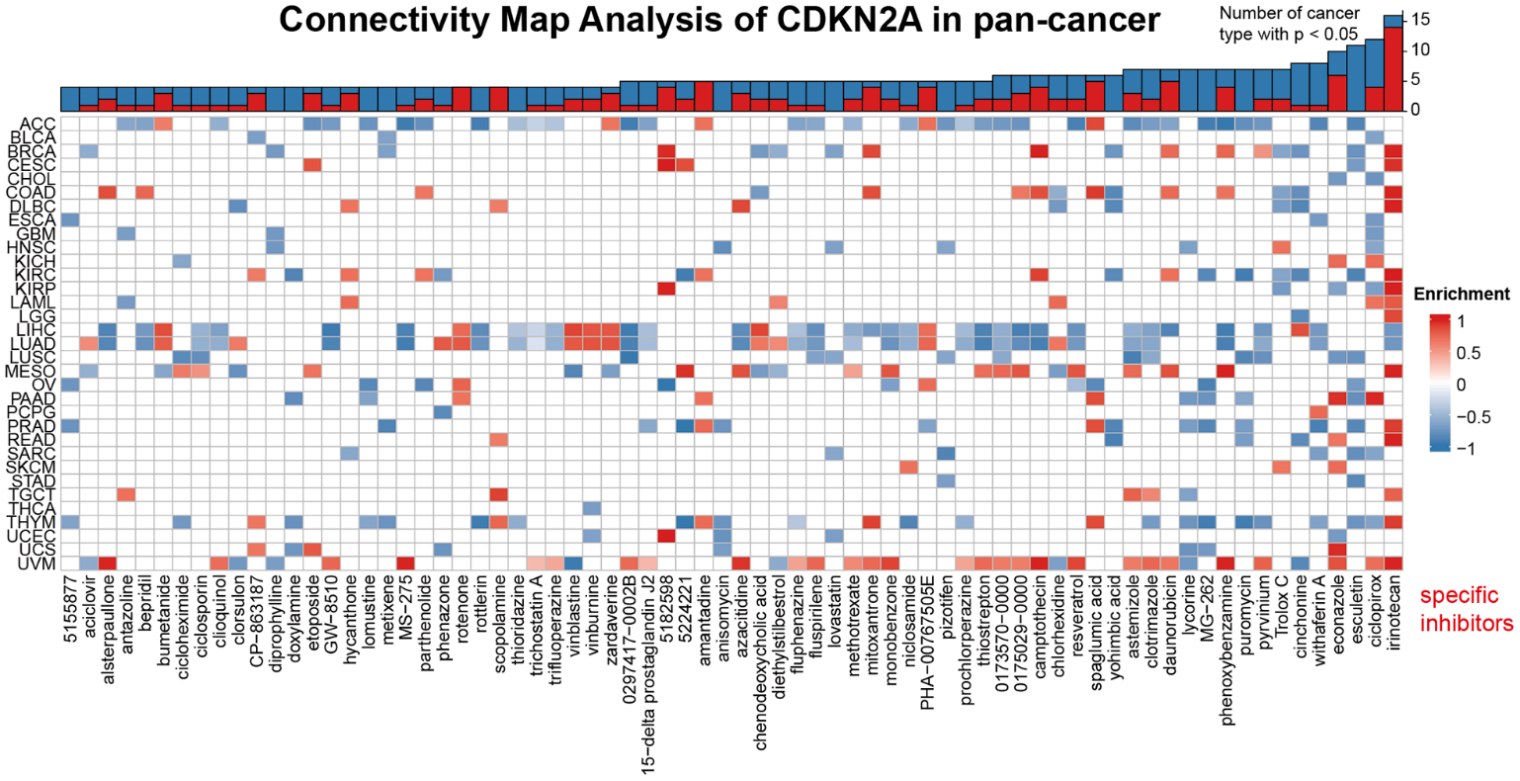
**Heat map represents the enrichment score (blue positive, red negative) for each drug in the CMap database for each cancer.** The components or drugs were sequentially decreased from right to left in the number of enriched cancers.

We demonstrated the potential components of targeted CDKN2A in pan-cancer in the form of heat maps using data downloaded from the CMap dataset (Figure 6). The heat map shows the drugs or components that may target CDKN2A in three or more cancer types. Irinotecan was highly abundant in 14 cancer types, especially at KIRC. Besides, ciclopirox, daunorubicin and amantadine are also enriched in at least 4 kinds of cancers. These drugs have been used in the prevention and immunotherapy of cancer. Irinotecan is used for immunotherapy of many cancers, especially advanced colon cancer and advanced pancreatic cancer [41]. Ciclopirox has been identified as a stable potential anticancer agent for a variety of cancer [42, 43] and has been shown to drive colorectal cancer cell death by activating ER [44]. Amantadine can be used as a clinical diagnostic test for lung and cancer [45]. Our results suggest that the existing clinical application of these drugs have more development space and application value, and their roles, potential targets and potential mechanisms in the occurrence and development of different cancers need to be further researched and explored.

## DISCUSSION

Tumor immunotherapy is the main trend and effective approach for tumor treatment nowadays [46, 47]. However, current immunotherapy is often targeted at a specific tumor, and only a part of cancer patients has a good prognosis due to the heterogeneity of tumor suppressive microenvironment in cancer patients [40]. A wide scope of response rates to tumor immunotherapy have been shown in tumors with low TMB, MSI loss, or even no PD-1/PD-L1 expression, suggesting that the immunotherapeutic response may be associated with other biomarkers [48]. Biomarkers for more accurate prediction of a patient's response to immunotherapy will enable individualized and precise immunotherapy for cancer patients. In our study, CDKN2A has been found to be a stable prognostic biomarker for pan-cancer and can effectively predict the response to ICI treatment. Our results could provide some clues for further research on the potential role of CDKN2A in tumor immunotherapy to explore and practical application.

We firstly assessed the expression levels of CDKN2A in pan-cancer based on TCGA and GTEx data. Almost all of TCGA cancers have a higher expression level of CDKN2A than normal tissues. And the associations between CDKN2A expressions and prognosis in different cancer patients were evaluated in this study. Strong prognostic role of CDKN2A in most cancers were observed, except TGCT. In particular, OS based prognostic analysis results suggest that CDKN2A is a risky predictor for the prognosis of tumors such as AAC, COAD, KICH, KIRC, LIHC, PCPG, THCA, UCEC, and UVM. The prognostic role of CDKN2A in cancers reflects the tumor suppressor function of it in cancers.

We performed gene set enrichment analysis of the hallmark gene sets from different tumor types to identify CDKN2A-associated cancer features. We found that the expression of CDKN2A was significantly associated with tumor immune-related pathways such as the TNFα signaling pathway via NFκB, IFN-α response, IFN-γ response, allograft rejection pathway, and inflammatory response. The above data reveal a potential association (TME) between CDKN2A expression and immune activation in the tumor microenvironment. That’s the primary motivation to search the immunotherapy response predictive efficiency of CDKN2A, as a biomarker under assessment. Interestingly, a recent study also found that non-squamous non-small cell lung cancer (NSCLC) tumor patients with deletion of 9p21.3, which includes CDKN2A sequence, showed worse clinical outcomes after pembrolizumab (anti-PD-1 therapy) [49].

Furthermore, we utilized the pan-cancer immune cell infiltration data in the TIMER2 database to conduct Spearman's correlation analysis, explored the correlation between CDKN2A expression and immune cell infiltrations, and further analyzed the relationship between CDKN2A expression and tumor immunity. In most TCGA cancers, CDKN2A is positively correlated with the degree of infiltration of CD4+ T cells, NKT cells, Tregs, neutrophils, and macrophages. However, how CDKN2A influences the TME via immune cells was still unknown, and this might be the key clues for the study involving the mechanisms.

We analyzed the correlation between CDKN2A and various immune factors in pan-cancer. We found a strong positive correlation between CDKN2A and most immunomodulatory factors in ACC, KICH, and LICH. To investigate the role of CDKN2A in predicting the function of immune checkpoint inhibitors (ICIs), we further evaluated the association of CDKN2A expression with TMB disease and MIS. Positive correlations with TMB were found in LGG, COAD, BLCA, thymus, THCA, STAD, SKCM, and PAAD. Furthermore, we demonstrated the potential component of targeted CDKN2A in pan-cancer using data downloaded from the CMap dataset. Which is of most interest, irinotecan was present in high correlation levels in 14 cancer types, particularly in KIRC. Irinotecan has been proved to be an effective chemotherapy drug to enhance the efficacy of several ICI therapy [50, 51]. Irinotecan prevents re-ligation of the DNA strand by binding to topoisomerase I-DNA complex and causes double-strand DNA breakage and cell death, but the role of CDKN2A in the underlying mechanisms involved might provide deeper insight for our further studies. According to our research, CDKN2A is a stable biomarker of tumor prognosis, which can effectively predict the response of immunotherapy and is closely related to tumor immune microenvironment. Our research can provide some clues for further research on the potential role of CDKN2A in tumor immunotherapy for exploration and practical application. And in the future, we will focus on the interaction between p16 protein in regulation of cancer immunity and associated underlying mechanisms.

## Supplementary Material

Supplementary Figures

Supplementary Table 1

Supplementary Table 2

Supplementary Table 3
