# Supplementary material for: The cell senescence regulator p16 is a promising cancer prognostic and immune check-point inhibitor (ICI) therapy biomarker
Source: Aging (Albany NY). 2023 Mar 23;15(6):2136–57. doi: 10.18632/aging.204601 (PMC10085592; doi:10.18632/aging.204601)
Supplement: Supplementary Figures [file aging-15-204601-s001.pdf]

SUPPLEMENTARY FIGURES

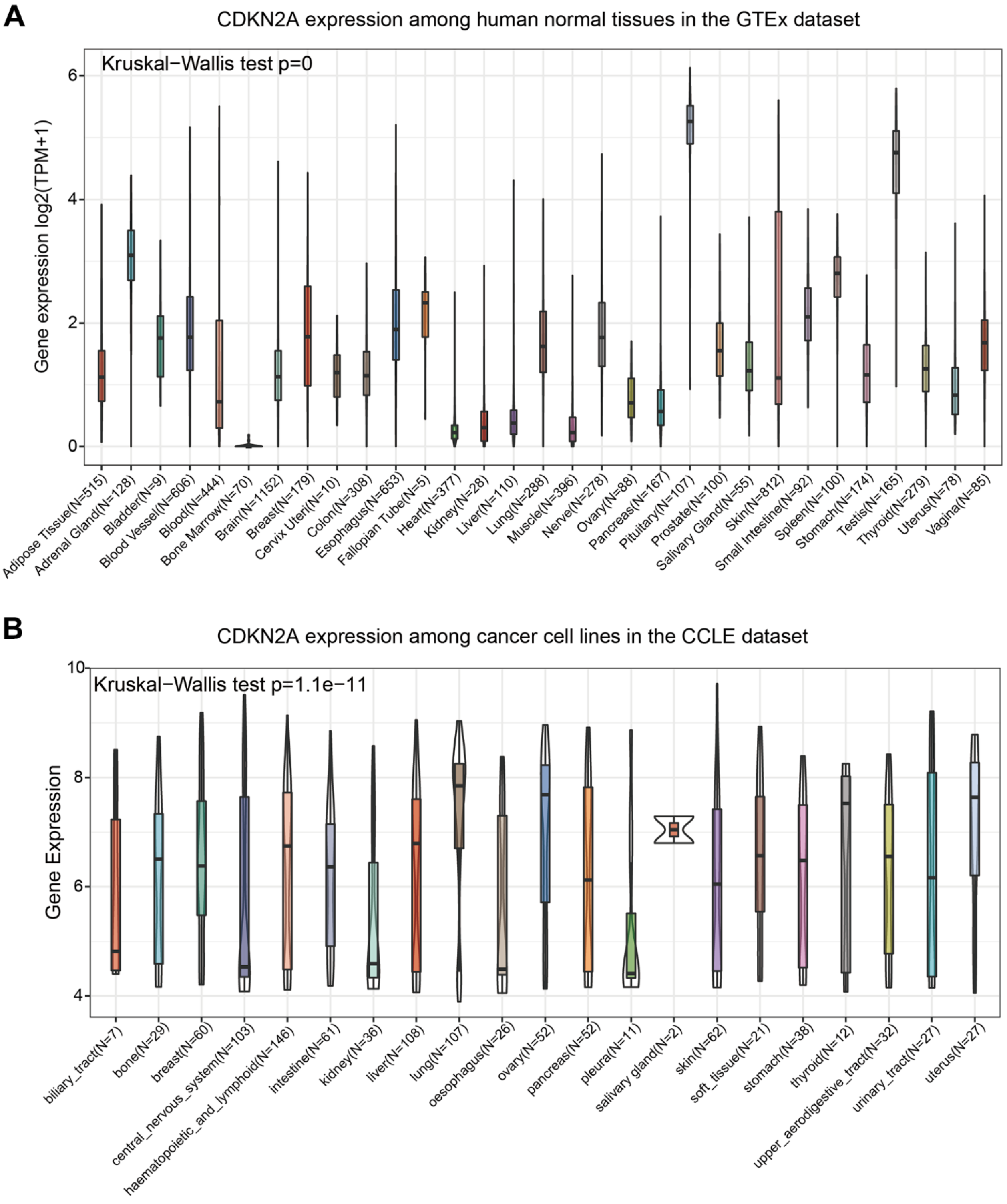

Supplementary Figure 1. Basic expression levels of CDKN2A across human normal tissues and cancer cells. (**A**) CDKN2A expression among human normal tissues in the GTEx dataset. (**B**) CDKN2A expression among cancer cell lines in the CCLE dataset.

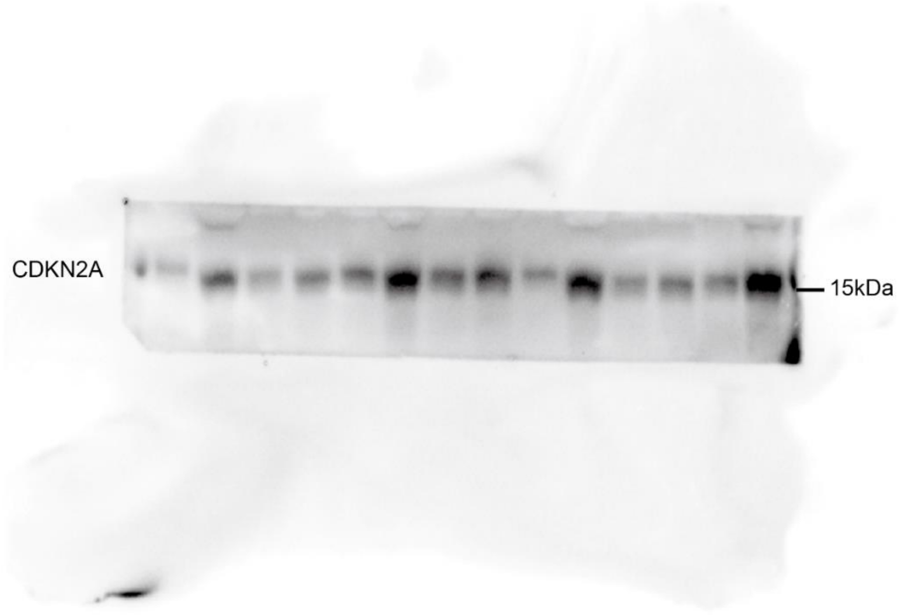

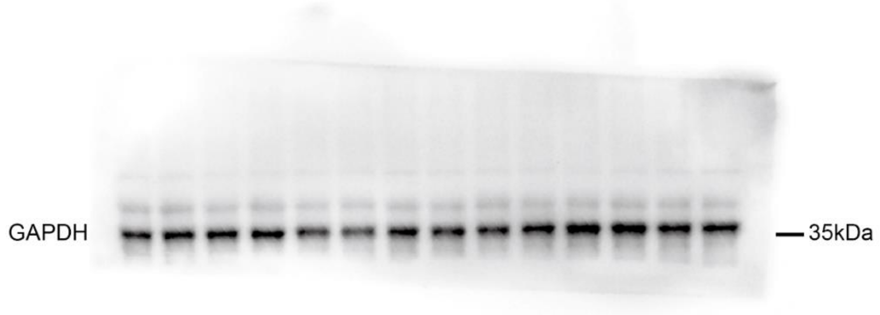

Supplementary Figure 2. Original Western blot bands of CDKN2A and GAPDH in 7 pairs of LGG samples.

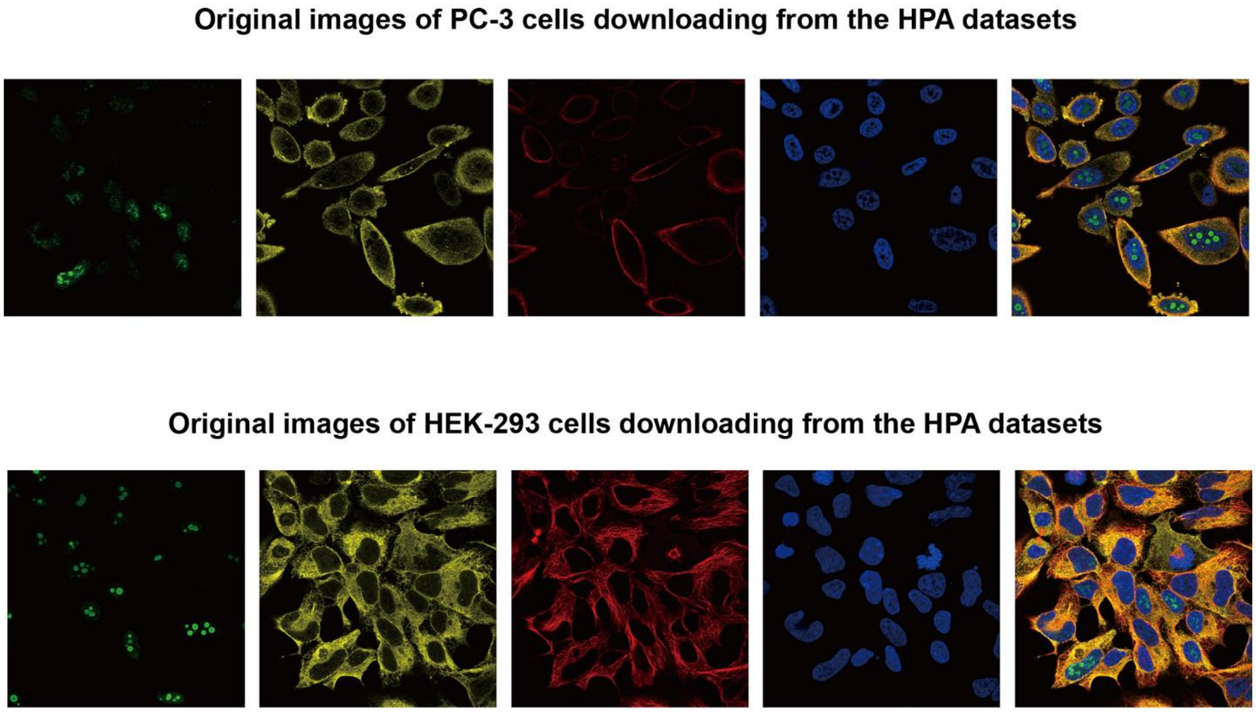

Supplementary Figure 3. Original images of immunofluorescent staining of CDKN2A in PC-3 and HEK-293 cells downloaded from the HPA dataset.

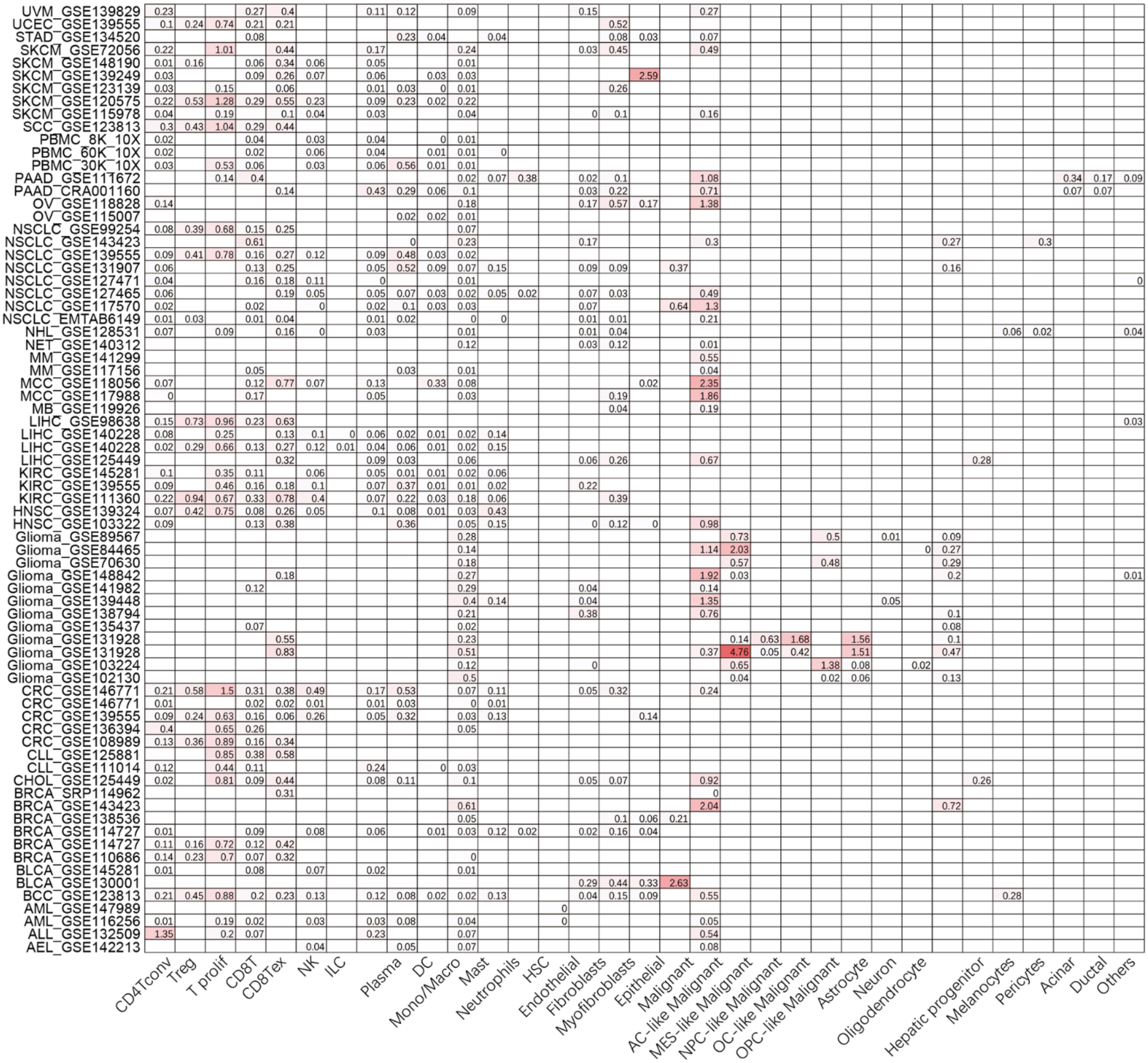

Supplementary Figure 4. The CDKN2A expression levels in different cell types in the single cell datasets.

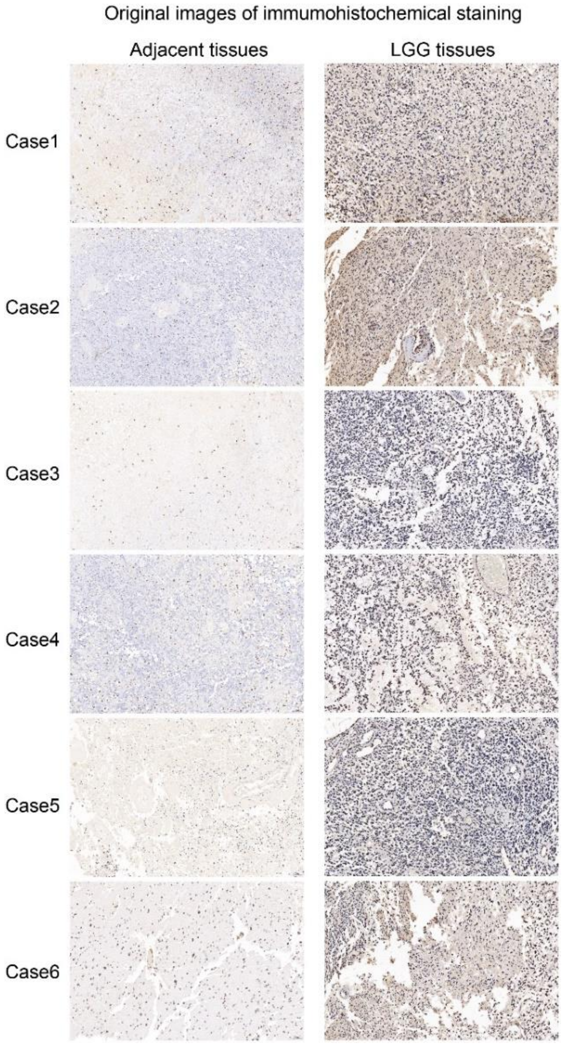

Supplementary Figure 5. Original images of immunohistochemical staining of CDKN2A in clinical LGG samples.

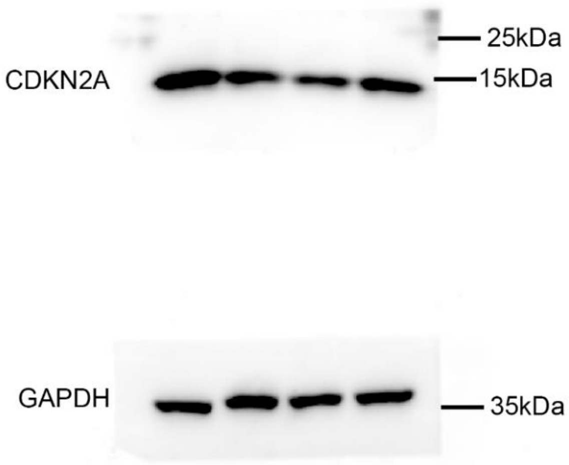

Supplementary Figure 6. Original Western blot bands of CDKN2A and GAPDH in si-CDKN2A transfected SW1088 cells.
